# Systems Integration of Biodefense Omics Data for Analysis of Pathogen-Host Interactions and Identification of Potential Targets

**DOI:** 10.1371/journal.pone.0007162

**Published:** 2009-09-25

**Authors:** Peter B. McGarvey, Hongzhan Huang, Raja Mazumder, Jian Zhang, Yongxing Chen, Chengdong Zhang, Stephen Cammer, Rebecca Will, Margie Odle, Bruno Sobral, Margaret Moore, Cathy H. Wu

**Affiliations:** 1 Protein Information Resource, Department of Biochemistry and Molecular & Cellular Biology, Georgetown University Medical Center, Washington, D.C., United States of America; 2 Virginia Bioinformatics Institute, Virginia Polytechnic Institute and State University, Blacksburg, Virginia, United States of America; 3 Social & Scientific Systems, Inc., Silver Spring, Maryland, United States of America; 4 Departments of Computer & Information Sciences and Biological Sciences, Delaware Biotechnology Institute, University of Delaware, Newark, Delaware, United States of America; Deutsches Krebsforschungszentrum, Germany

## Abstract

The NIAID (National Institute for Allergy and Infectious Diseases) Biodefense Proteomics program aims to identify targets for potential vaccines, therapeutics, and diagnostics for agents of concern in bioterrorism, including bacterial, parasitic, and viral pathogens. The program includes seven Proteomics Research Centers, generating diverse types of pathogen-host data, including mass spectrometry, microarray transcriptional profiles, protein interactions, protein structures and biological reagents. The Biodefense Resource Center (www.proteomicsresource.org) has developed a bioinformatics framework, employing a protein-centric approach to integrate and support mining and analysis of the large and heterogeneous data. Underlying this approach is a data warehouse with comprehensive protein + gene identifier and name mappings and annotations extracted from over 100 molecular databases. Value-added annotations are provided for key proteins from experimental findings using controlled vocabulary. The availability of pathogen and host omics data in an integrated framework allows global analysis of the data and comparisons across different experiments and organisms, as illustrated in several case studies presented here. (1) The identification of a hypothetical protein with differential gene and protein expressions in two host systems (mouse macrophage and human HeLa cells) infected by different bacterial (*Bacillus anthracis* and *Salmonella typhimurium*) and viral (orthopox) pathogens suggesting that this protein can be prioritized for additional analysis and functional characterization. (2) The analysis of a vaccinia-human protein interaction network supplemented with protein accumulation levels led to the identification of human Keratin, type II cytoskeletal 4 protein as a potential therapeutic target. (3) Comparison of complete genomes from pathogenic variants coupled with experimental information on complete proteomes allowed the identification and prioritization of ten potential diagnostic targets from *Bacillus anthracis*. The integrative analysis across data sets from multiple centers can reveal potential functional significance and hidden relationships between pathogen and host proteins, thereby providing a systems approach to basic understanding of pathogenicity and target identification.

## Introduction

The NIAID (National Institute of Allergy and Infectious Diseases) Biodefense Proteomics program, established in 2004, aims to characterize the pathogen and host cell proteome by identifying proteins associated with the biology of microbes, mechanisms of microbial pathogenesis and host responses to infection, thereby facilitating the discovery of target genes or proteins as potential candidates for the next generation of vaccines, therapeutics, and diagnostics [1]. The program includes seven Proteomics Research Centers (PRCs) conducting state-of-the-art high-throughput research on pathogens of concern in biodefense and emerging/reemerging infectious diseases, as well as a Biodefense Resource Center for public dissemination of the pathogen and host data, biological reagents, protocols, and other project deliverables.

The PRCs work on many different organisms, covering bacterial pathogens (*Bacillus anthracis*, *Brucella abortus*, *Francisella tularensis*, *Salmonella typhi*, *S. typhimurium*, *Vibrio cholerae*, *Yersinia pestis*), Eukaryotic parasites (*Cryptosporidium parvum*, *Toxoplasma gondii*), and viral pathogens (Monkeypox, SARS-CoV, Vaccinia). The centers have generated a heterogeneous set of experimental data using various technologies loosely defined as proteomic, but encompassing genomic, structural, immunology and protein interaction technologies, as well as more standard cell and molecular biology techniques used to validate potential targets identified via high-throughput methods. In addition to data, the PRCs have provided biological reagents such as clones, antibodies and engineered bacterial strains, other deliverables include standard operating procedures (SOPs) and new technologies such as instrumental methods and software tools and finally publications related to all of these activities.

Consequently, there were a number of unique challenges facing the Resource Center: (i) how to coordinate with the seven PRCs with various pathogens, technologies, processes, and data types; (ii) how to provide seamless integration of three institutions that make up the Resource Center; and (iii) how to provide timely and effective dissemination of newly discovered information to the user community. In particular, due to the breadth of the program, the potential user community is quite broad, from technology or informatics experts who may want to reanalyze the data or develop better algorithms, to a wide group of biomedical scientists who are interested in mining the data for their own studies or just finding new information on a protein or gene of interest quickly and easily.

Accordingly, we developed a set of functional requirements early in the Biodefense Resource Center development: (i) to implement a center-specific submission protocol and data release plan for timely dissemination, (ii) to promote data interoperability, adopting common standards (such as HUPO Proteomic Standards Initiative [2], [3], [4]), defining a core set of metadata with mapping to controlled vocabularies and ontologies, recommending preferred IDs for gene/protein mapping, and (iii) to provide value-added annotation to capture key findings and integration of the data with related resources for functional interpretation of the data. Available online at http://proteomicsresource.org, the architecture, initial content and general features of the Biodefense Proteomics Resource were briefly described elsewhere [5]. A breakdown of the Resources content by organism, PRC and other criteria can be seen at: http://www.proteomicsresource.org/Resources/Catalog.aspx. Tutorials and help are provided on the website: http://www.proteomicsresource.org/Resources/Tutorials.aspx , http://proteininformationresource.org/pirwww/support/help.shtml#30

The objective of this study is to provide a systems approach to the study of pathogen-host interactions, connecting the various types of experimental data on genomics, proteomics and host-pathogen interactions with information on pathways, regulatory networks, literature, functional annotation and experimental methods. Having most of this information accessible in one place can facilitate knowledge discovery and modeling of biological systems. Like many problems in data integration, it is easy to know the general outline of what we want, but often much harder to implement and navigate the information, especially if the original data crosses disciplinary, laboratory and institutional boundaries. Here we describe in detail a protein-centric approach for systems integration of such a large and heterogeneous set of data from the NIAID Biodefense Proteomics program, and present scientific case studies to illustrate its application to facilitate the basic understanding of pathogen-host interactions and for the identification of potential candidates for therapeutic or diagnostic targets. Several scientific use cases are presented that illustrate how one can search varied experimental data from different laboratories and even ones researching different infectious organism and their hosts to make potentially useful connections that could lead to new hypotheses and discoveries.

## Methods

### Biodefense Resource Center Infrastructure

**Figure 1:**
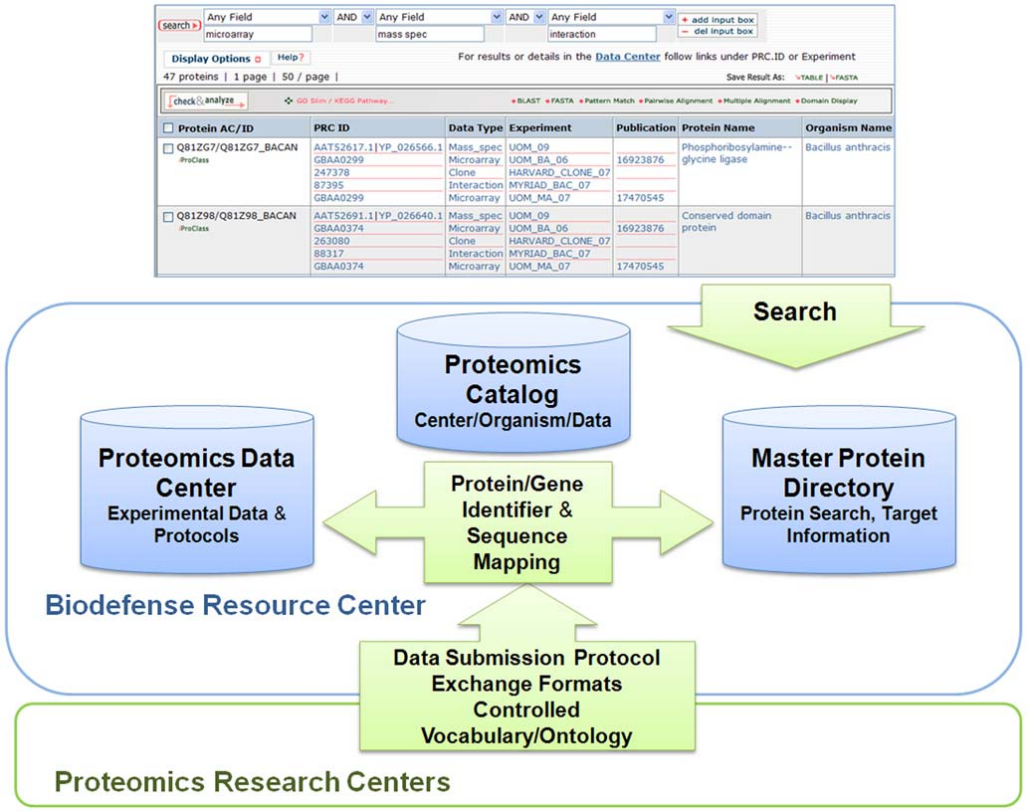
General infrastructure and information flow of the Biodefense Resource Center. The Master Protein Directory provides protein-centric data integration, search and analysis capabilities. The Proteomics Catalog houses web pages with information on the PRCs, the NIAID Proteomics Program and project related publications. The Proteomics Data Center contains complete experimental data and protocols for each PRC data set in relational databases but is not integrated in the protein-centric manner. All databases are linked on the web.

Based on the functional requirements of the Resource Center, we developed a bioinformatics infrastructure for integration of PRC deliverables (Figure 1). In our workflow, multiple data types from PRCs are submitted to the center using a data submission protocol and standard exchange format, with the metadata using controlled vocabulary whenever possible. For functional interpretation of the data, we then map the gene and protein data based on identifier (ID) mapping or if necessary using peptide or sequence mapping to proteins in our data warehouse described below. All of the databases, along with information on the PRCs and organisms under study are listed in the proteomics catalog accessible from the web portal.

### Protein-Centric Data Integration

The key design principal in the Resource Center is protein-centric data integration. Here the diverse experimental data are integrated and presented in a protein-centric manner where information is queried and presented via common proteins and connected to experimental data and the network of protein attributes, including information on the encoding genes, protein families, pathways, functions and more. In practice a protein-centric approach works well as proteins occupy a middle ground molecularly between gene and transcript information and higher levels of molecular and cellular structure and organization. Proteins are often the functional molecules in biological processes described by pathways, molecular interactions and other networks. Protein families, in turn, have proven to be invaluable in studying evolution and for inferring and transferring functional annotation across species.

### Master Protein Directory

Underlying the protein-centric data integration is a data warehouse called the Master Protein Directory (MPD) where key information is extracted from the primary data and combined for rapid search, display and analysis capabilities. The MPD is built on the data and capabilities of iProClass [6] a warehouse of protein information, which in turn is built around UniProtKB [7] but supplemented with additional sequences from gene models in RefSeq [6] and Ensembl [7] and additional annotation and literature from other curated data resources such as Model Organism Databases [8], [9], [10], [11], [12], [13] and GeneRIF [14]. The biodefense data are essentially additional data fields added to a subset of iProClass entries to create the MPD.

Currently the MPD defines and supports information from the following types of data produced by the PRCs: Mass Spectrometry, Microarray, Clones, Protein Interaction, and Protein Structure. More data types or attributes may be added in the future if needed. Supplemental table S1 shows the common and unique fields used in the MPD for each data type. An advantage of the data warehouse design is that, if needed, additional fields can be extracted from the primary data and easily added as new attributes without greatly altering the existing database design or query mechanisms. The MPD data is stored in an Oracle database along with iProClass data. For data exchange, an XML file and schema for the current MPD are available for download at:

ftp://141.161.76.88/pub/proteomics_ftp/rc/MD_data/iproclass_mpd.dtd

ftp://141.161.76.88/pub/proteomics_ftp/rc/MD_data/iproclass_mpd.xml

ftp://141.161.76.88/pub/proteomics_ftp/rc/MD_data/iproclass_mpd.xsd

The MPD including the website and FTP files is updated every 3 weeks in conjunction with iProClass or whenever new PRC data is released.

### Protein Mapping Process

The various Proteomics Research Centers all used different sources and identifiers for the nucleotide and protein sequences in their analysis pipelines and occasionally would change sources depending on the experiment. This is a common problem encountered when attempting to combine data across research laboratories unless identical sequence databases, processes, platforms and organism names are used. Examples of database identifiers used include Genbank/EMBL/DDBJ accessions and locus tags, UniGene accessions, RefSeq accessions, IPI accessions, NCBI gi numbers and IDs unique to a sequencing center or organism-specific database. The first step was to map all experimental results to a common representation of a protein. This was achieved by mapping all protein and gene IDs and names to iProClass proteins. The majority of the mapping using IDs from public resources was done using mapping services and tables provide on the Protein Information Resource (PIR) web site (http://proteininformationresource.org/pirwww/search/idmapping.shtml) and FTP site (ftp://ftp.pir.georgetown.edu/databases/iproclass/). However, some mapping problems needed to be addressed either by automated rules, direct sequence comparisons or manual analysis and annotation.

### Problems and Solutions

Mapping difficulties fell into 4 categories: 1) **One-to-many mappings**: a common problem, especially when eukaryotic host proteins derived from alternate splicing or viral polyproteins are involved. UniProtKB usually merges information on alternate splice forms or polyproteins, which helped minimize this problem for our purposes, but in cases where multiple mappings exist, we selected as most informative the entry in the manually reviewed UniProtKB/SwissProt section; if no SwissProt entry was found, the longer sequence in UniProtKB/TrEMBL section was selected. Users could always find the alternate mappings via the iProClass related sequences link on the MPD entry page to precompiled BLAST results on all iProClass sequences. 2) **Retired sequences**: genomic sequences from databases such as RefSeq, IPI, or UniGene are not static and, as information changes, some gene predictions and translations are retired with each new build. Retired sequences often required manual mapping by a curator to match original gene or peptide results to current protein sequences. Primarily UniParc (UniProt sequence archive) [15] was used for this purpose. 3) **Protein sequences not available in iProClass or any public repository**: this occurred most often with *Toxoplasma gondii* whose genome sequencing was still in progress and stable builds were not yet available. However, the problem also occurred in some well-characterized organisms like *Vibrio cholera* and *Bacillus anthracis*. Several data sets contained information on annotated but not translated pseudogenes. In the case of *Vibrio cholera*, 21 of 48 pseudogenes cloned and sequenced by the Harvard Institute of Proteomics did not contain the annotated point mutation or frame shift and appeared to produce full-length proteins [16]. In the case of *Bacillus anthracis*, microarrays containing probes for 82 of 192 annotated pseudogenes also showed significant changes in RNA expression in response to infection or other treatments [17], [18], [19]. In these cases, new database entries were created in the MPD to house the results. 4) Alternate species or strain representations: several experimental data sets reported sequence identifiers for strains or variants other than the one used in the experimental sample. This is not an uncommon situation as the genetically most characterized variant is often an attenuated laboratory strain while the more virulent strains are either not yet sequenced or the sequence is of lower quality. Often microarray chips or mass spectrometry databases are designed using the best available sequence from the research strain, yet then use RNA and protein samples from another similar virulent strain. The question here was what organism strain to map to and represent on our website: the strain the RNA or protein the sample came from, or alternatively, the strain that matched the identifier and sequence used in the research to detect the RNA or protein. For the MPD we chose to map to the sequence identifier reported in the data files and related publications, with the additional virulent strain information noted in the results summary.

### Search Design

#### Data Mining Design Goals

In consultation with NIAID and PRCs within the project's Interoperability Working Group, we developed 3 goals for data mining. 1) All project data and other deliverables should be available via browsing and simple keyword searches. 2) The data and information provided by the resource should be sufficient to allow a skilled researcher to download and reanalyze or mine the data for additional information. 3) Our target user was a biomedical scientist not expert in the technology used to produce the data or in bioinformatics, thus the data, procedures, publications and general results and conclusions of an analysis should be relatively easy to find on the project website for someone not familiar with the details of the particular technologies used to generate it.

To allow both simple keyword searches and also Boolean searches of the project data, we did the following: 1) We included in the MPD only “validated” results that were determined by the research centers to be significant using their methods. To do otherwise would confuse users not familiar with the technology and how results are filtered. Results that fell below the significance threshold used by the research center were made available via download of data sets at the FTP site. 2) “RAW” unprocessed machine specific data would be stored by the PRC and available on request. 3) To facilitate and simplify searches across laboratories and data types, we omitted most data type or analysis specific numerical values and statistics from the general MPD search and display. These numerical values are usually platform, laboratory and method dependent and cannot easily be used to compare across datasets so including them might be confusing to users. Instead, we focused on providing simple, yet powerful, queries of experimental summaries where a user can query if a gene/protein was presented in the results and, in some cases, if it showed a reported increase or decrease in expression or accumulation based on the PRC's criteria. Once a set of proteins of interest is identified, a user can then drill down to view the specific experimental values and methods employed to generate the particular dataset. Links to details in the publications are provided and full data are available via FTP. Since all protein attributes are included as search options, one can query beyond simply protein names, accessions or project data and search pathways, protein families, Gene Ontology (GO) terms, database cross-references and many other attributes, providing many powerful options to the users.

To provide a robust text search for the website, we used the PIR text indexing system [20] in which over 100 text fields and unique identifiers from the MPD database are indexed using Callable Personal Librarian (CPL) [21] which supports fast exact text search, substring & wildcard text search, range search and Boolean searches. Entry indexing and retrieval is supported by Oracle.

#### I: Unstructured keyword search

**Figure 2:**
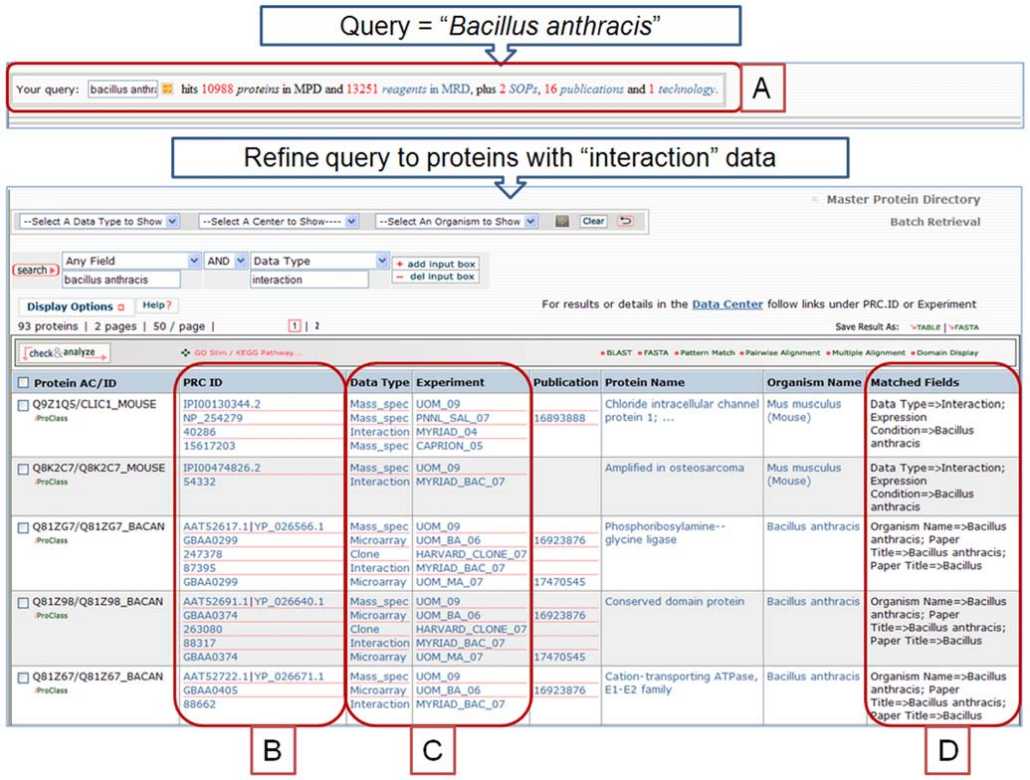
Unstructured Keyword Query. A) The keyword “bacillus anthracis” hits results for project related proteins, SOPs, literature and technologies. The query can then be refined further by using specific fields to form a structured query. Here the search is restricted to return only proteins with interaction data. B) Multiple gene and protein IDs provided by the PRCs are merged into one representation of the protein. C) All data types and experimental datasets from different centers are listed together. D) Matched fields are displayed to aid users in formulating queries. To reproduce these queries use this URL: http://pir.georgetown.edu/cgi-bin/textsearch_cat.pl?search=1&field0=all&query0=bacillusanthracis&andor1=and&field1=DATATYPE&query1=interaction .

A simple keyword search was implemented on every page of the resource's website. This searches all fields in the MPD. To further facilitate searches, the protein name field is supplemented by also searching the BioThesaurus [10] containing all gene and protein name synonyms and textual variants for each protein from over 35 data sources. In addition, the default option searches all text from the PubMed abstract for all project publications, an abstract of each technology and all text in SOPs. The text indexed for publications, technologies and SOPs was annotated with additional standard keywords to facilitate searches. Figure 2 shows the results of a simple keyword search with hits for proteins, reagents, publications, technologies and SOPs.

#### II: Structured text search

The MPD database contains over 100 fields derived from iProClass and Proteomics Research Center's data. Currently 75 of these fields are available for individual searches and can be combined with Boolean operators as seen in some of the use case examples in this paper.

The protein-centric search results are presented in a customizable tabular format where users can add or delete columns. Currently 62 fields can be customized. The tabular display has two modes: 1) a default mode which displays fields common to all the supported data types and 2) a data type specific mode which restricts the results to a particular data type and displays fields specific to that data type. See supplemental table S1 for a list of data type specific fields. Additional filters for Proteomic Research Center and Organism are available as pull down menus to aid browsing and viewing the results of queries. Examples of these functions are illustrated in the scientific use cases below and in help pages and tutorials available on the website. http://www.proteomicsresource.org/Resources/Tutorials.aspx, http://proteininformationresource.org/pirwww/support/help.shtml#30.

## Results

### Data Base Content – Browsing Pathogen-Host Omics Data

The resource currently contains information on 35,112 proteins from 58 datasets, 35,819 reagents, 75 SOPs, 31 technologies and 88 manuscripts. Table 1 shows statistics on proteins in the MPD. Currently ∼30% of the proteins are uncharacterized in that they are called either “Uncharacterized” or “Hypothetical” and have no other functional annotations or functional domains. Of these uncharacterized proteins, ∼85% have experimental data, such as mass spectrometry, microarray, or protein interactions, associated with them. The remaining 15% of uncharacterized proteins are available as full length clones for further research. Though the program is focused on pathogen proteins, about 32% of the proteins are host proteins from mouse or human, as cell lines or tissue samples from both these organisms were used as infection models for multiple pathogens.

**Table 1:** Classification of MPD proteins.

Source	Protein Count
All Organisms	35,112
*Mus musculus* (Mouse)	7,823
* Toxoplasma gondii*	6,678
* Bacillus anthracis*	5,854
* Vibrio cholerae*	3,732
*Homo sapiens* (Human)	3,526
* Salmonella typhimurium*	3,406
* Salmonella typhi*	2,061
* Brucella abortus*	944
* Cryptosporidium parvum*	609
Vaccinia virus	161
Monkeypox virus	130
*Francisella tularensis*	62
*Yersinia pestis*	75
Human SARS virus	6
Mass Spec Data	25,289
Microarray Data	7,031
Interaction Data	1,363
New Structures, (Domains)	6 (15)
Sequenced Clones	9,074
Uncharacterized Proteins	10,637
Proteins in Pathways	9,988
Classified in Families	24,953
With GO Terms	22,265
With Bioinformatics Resource Center Links	18,165
With Immune Epitope DB links	583

The content of the MPD as of July 2009. Numbers represent the total number of proteins that meet the listed criteria, for example, have one or more GO term or pathway or link associated with the protein.

### Web-based Protein Search – Searching Pathogen-Host Omics Data

#### Simple Keyword Search

Due to the popularity of internet searches, support of unstructured keyword queries, even for structured data, has become critical for any web site. To support this feature, the default protein-centric search returns results for all project deliverables, data, reagents, protocols, technologies and publications. An example is shown in Figure 2 where a single keyword search “bacillus anthracis” finds 10,988 pathogen and host proteins with Mass spec, microarray or protein interaction data and also 13,251 reagents in the Master Reagent Directory (mostly ORF and Y2H clones and mutant bacterial strains of *Bacillus anthracis*), 2 SOPs, 16 project publications and 2 technologies. The matched fields column allows users to refine their queries and construct simple Boolean searches.

### Biological Use Case Examples – Analyzing Pathogen-Host Omics Data

#### Example I: Integrative Analysis

**Figure 3:**
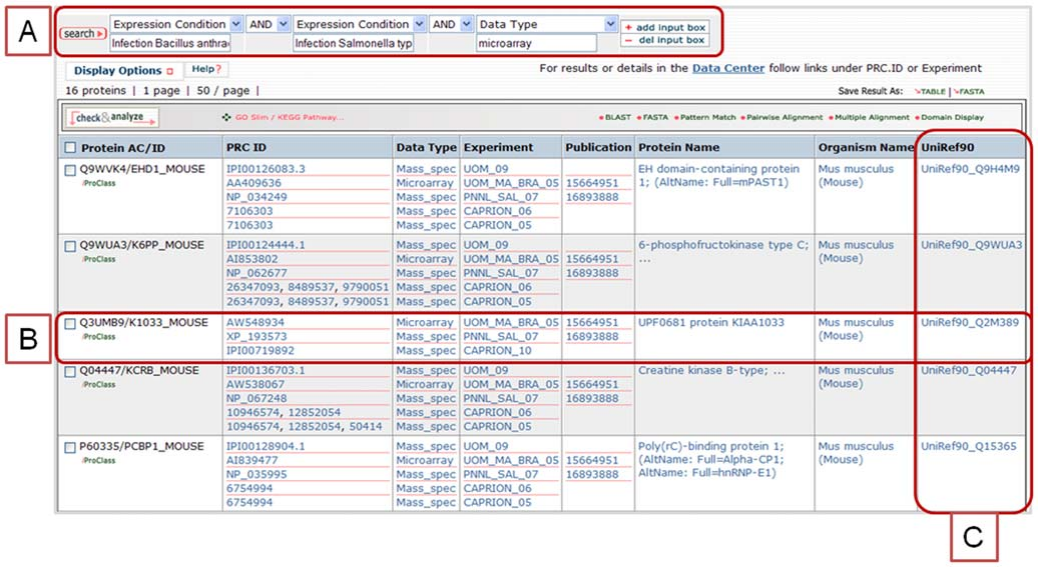
Structured queries across infectious agents and data types. A) Search for proteins detected in studies of *Bacillus anthracis* AND *Salmonella typhimurium* infection that include microarray experiments, finds 16 host proteins from mouse macrophage cell lines. B) Results include a large uncharacterized protein K1033_MOUSE. C) A customizable web interface allows users to add and view related information on pathways of protein families. To reproduce these queries use this URL: http://pir.georgetown.edu/cgi-bin/textsearch_cat.pl?search=1&field0=EXPC&query0=InfectionBacillusanthracis&andor1=and&field1=EXPC&query1=InfectionSalmonellatyphimurium&andor2=and&field2=datatype&query2=microarray .

Structured fields allow Boolean queries across organisms, data types and laboratories. Figure 3 shows a query where we make use of the controlled vocabulary in the “expression condition” field and searched for common host proteins detected in studies of *Bacillus anthracis* and *Salmonella typhimurium* infection in mouse macrophage cell lines. Currently 222 proteins meet these criteria, mostly from mass spectrometry studies by PNNL and the University of Michigan; however, if we further restrict the search to include only those also detected in microarray experiments, we find 16 proteins with mass spec data from *S. typhimurium* infections at one research center, and mass spec and microarray studies done using *B. anthracis* at another research center. From the customizable results display shown in Figure 3 we can view summary information on the proteins detected. Full details on the protein and individual experimental results are available via links [5].

Some benefits of the protein-centric mapping approach are visible in the default display. 1) Minimizing redundancy, the column PRC ID shows the different identifiers from different databases used by the research centers. In the case of one protein Q9WUA3/K6PP_MOUSE 6-phosphofructokinase type C (EC 2.7.1.11), a total of 9 identifiers from 4 different databases (Unigene, RefSeq, IPI, nr) were reported in the research results that all represent either the gene or protein sequence for this single mouse protein. 2) Discovery of additional experimental information from other studies. Fifteen of the sixteen proteins found in the query also have mass spec data from Caprion Proteomics, as indicated in the ‘experiment’ column by Caprion_05, 06 and _10. Caprion is studying *Brucella abortus* and using a similar mouse macrophage model. Currently, only the data for uninfected macrophages is available from Caprion. Additional data on *Brucella* and mouse proteins from bacterial infected macrophages should be included in future releases. From the results display, one can follow links to view the iProClass protein report with executive summaries of the results from the PRCs and information collected from over 100 public resources or drill down to view the specific peptides or expression values seen in these studies, read publications and methods about the experiments or download the data for additional analysis.

**Figure 4:**
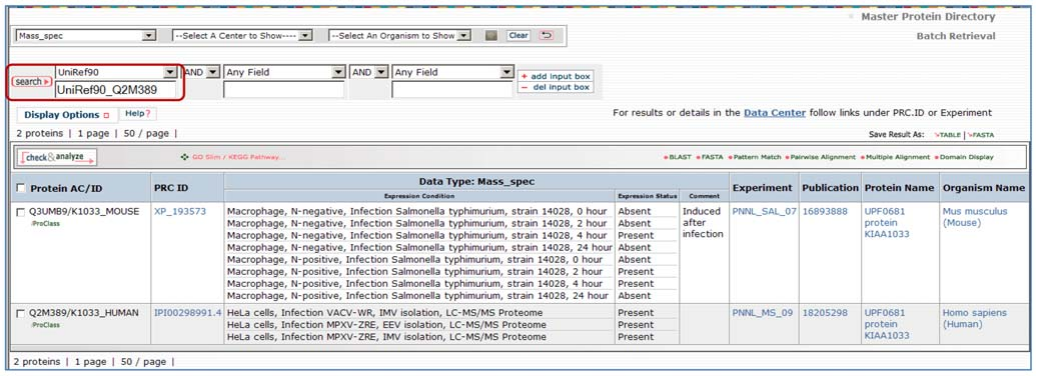
Queries on cluster or family information. Queries on cluster or family information can discover related information across laboratories and host pathogen systems. Using the UniRef90 cluster ID for K1033_MOUSE, identified in Figure 3, to query for all proteins with at least 90% identity, we find the human homolog K1033_HUMAN was detected in HeLa cells infected with Vaccinia and Monkypox virus [12].

With a comprehensive protein data warehouse, one can also broaden the search for relevant data and information from related organisms using protein cluster or family information. In Figure 4 we illustrate this by selecting one protein, K1033_MOUSE, an uncharacterized protein seen in three datasets from infected mouse macrophages. Using the UniRef90 cluster ID [22] to query for all proteins with at least 90% identity and no gaps, we find the human homolog K1033_HUMAN was detected in HeLa cells infected with vaccinia and monkypox virus. If we do a batch retrieval using Uniref90 IDs from all 16 original mouse proteins, we find 12 human homologs were also detected in studies of orthopox infection (not shown). The identification of a hypothetical protein with differential gene and protein expressions in two host systems (mouse macrophage and HeLa cells) infected by different bacterial (*Bacillus anthracis* and *Salmonella typhimurium*) and viral (orthopox) pathogens suggest that this protein should be prioritized for additional analysis and functional characterization.

#### Example II: Combining data from different studies

Discovering and comparing experimental results across laboratories and data types can help lead to new hypotheses for further experimentation [23], [24], [25]. Although different laboratories use different sample preparation, detection and analysis techniques making some direct comparisons difficult, having the data together in one place allows queries and comparisons between proteins and gene sets to be combined and additional analysis undertaken.

**Figure 5:**
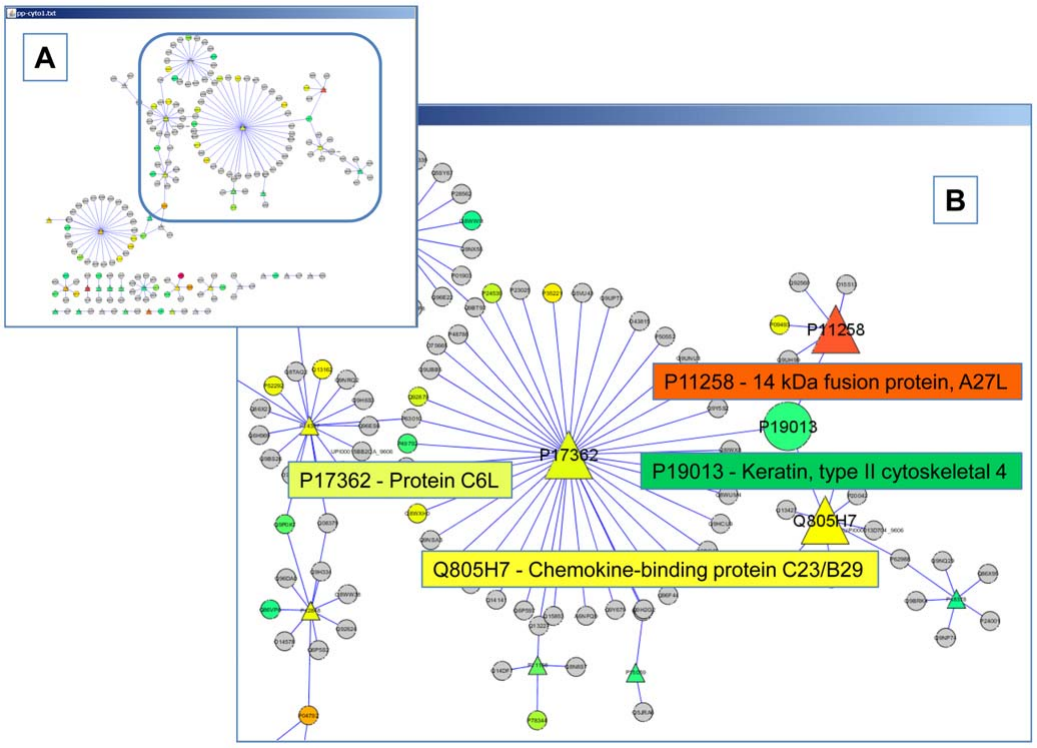
Vaccinia virus, human protein interaction network. A) Triangular nodes represent Vaccinia proteins, round nodes human proteins. Colors represent relative abundance as determined by peptide counts. Grey nodes represent no peptide data, green to yellow to orange to red represent increasing spectral counts in the range of 3 to 543. B) The three interactions, discussed in the text. The host protein (Keratin, type II cytoskeletal 4, UniProt: P19013) and three viral proteins (P11258 - 14 kDa fusion protein, A27L; Q805H7 - Chemokine-binding protein C23/B29; P17362 - Protein C6L).

In this example, data from different labs and data types are combined for further analysis. A query of the MPD on data type  =  “interaction” AND organism name  =  “virus” finds 33 vaccinia virus proteins with interactions with human proteins determined by Myriad Genetics. Browsing the results display shows that 25 of the proteins were also seen in mass spec work published by PNNL [26]. By combining the two experimental data sets Experiment  =  “MYRIAD_05” AND “PNNL_MS_09”, we find 83 virus and human proteins with both mass spec and interaction data from each laboratory. Further investigation into the experimental details shows that a protein interaction network was determined by Myriad Genetics using a yeast-two hybrid assay to screen viral bait proteins against a library of human prey proteins cloned from different tissues. The mass spec data from PNNL was obtained from viral preparations isolated from infected human HeLa cells. The work from PNNL contained quantitative information in the form of spectral counts and Accurate Mass Tag [27] intensities, downloadable from the project FTP site. We combined all the vaccinia plus human interaction data with peptide counts for each protein and visualized the results using Cytoscape [28], [29]. The complete network of results is shown in Figure 5A.

Various methods have been tried and compared for filtering large intra-species interaction networks to limit false positives and to select the biologically relevant interactions [30], [31], [32], [33], [34], [35]. Relatively little has yet been done for inter-species pathogen-host networks. Several common factors that have proved useful in other studies are 1) evaluating network hubs with many interactions and 2) using correlations between interacting pairs with similar gene expression patterns. For this small interactome, we looked for relatively abundant proteins associated with multiple interactions. We identified a single human protein interacting with three viral proteins, three being the largest number of viral interactions seen with a single host protein in this data set. The interactions are shown in Figure 5B. The host protein Keratin, type II cytoskeletal 4 (P19013) interacts with three viral proteins (P11258 - 14 kDa fusion protein, A27L; Q805H7 - Chemokine-binding protein C23/B29; P17362 - Protein C6L). The 14 kDa fusion protein A27L is the most abundant protein seen in this data set and participates in virus penetration at during cell fusion [36], [37]. A27L facilitates initial attachment to cells by binding to glycosaminoglycans [38]. A27L is found in all orthopoxviruses and has no cellular or entomopoxvirus homologs. Additional viral proteins involved in attachment (D8L, H3L) [39], [40] and fusion (F9L, I2L) [41], [42] were not observed. The Chemokine-binding protein C23L belongs to a family of poxvirus chemokine-binding proteins that mimic the chemokine response and prevent activation and chemotaxis of leukocytes [43], [44]. Protein C6L belongs to a family of poxvirus paralogs that may function as toll-like receptor inhibitors based on homology to A52R [45], [46], [47]. Thus, this protein may modulate Toll/IL-1R signaling, resulting in a diminished host immune response and enhancing viral survival.

P19013 - Keratin, type II cytoskeletal 4, the host protein, has tissue specificity in the suprabasal layer of the stratified epithelium of the esophagus, exocervix, vagina, mouth and lingual mucosa, and in cells and cell clusters in the mucosa and serous gland ducts of the esophageal submucosa [48]. Transgenic knockout mice have shown Keratin, type II cytoskeletal 4 to play an important role in maintaining normal epithelial tissue structure [49]. Keratin 4 in human saliva has been shown to interact with the protein Srr-1 localized on the surface of *Streptococcus agalactiae* and to play a critical role in colonization of this bacterial pathogen [50], [51].

Little is known about the mechanisms by which poxviruses attach to and enter host cells. No receptor for virion attachment on the host cell surface has been found. Poxvirus infection can occur through interaction with human as well as mice airway epithelia, [52], [53] we propose that the protein interactions outlined above may represent some of the initial interactions between host and pathogen. Thus they represent potential therapeutic targets for further investigation. This is the first report describing the interaction of a poxvirus protein with a host Keratin, type II cytoskeletal 4 protein.

#### Example III: Screening for Pathogen Specific Target Proteins

Unequivocal identification of pathogens is important so that adequate counter measures can be taken. Currently over 700 pathogenic and non-pathogenic bacteria have been completely sequenced. The availability of sequence data allows identification of proteins that are unique at different taxonomic levels, thus providing a means to begin to distinguish pathogenic from non-pathogenic species. However, if the initial screening depends on sequence data alone, the list of potential targets for laboratory validation can be relatively long; by supplementing sequence results with experimental data one can prioritize the target list for validation in the laboratory. We used such an approach by computationally screening potential targets using CUPID [54], PRC data and other computational means to produce a list of potential targets.

Identifying species-specific proteins can be done with confidence when multiple species and strains have been sequenced as is the case with *Bacillus anthracis*. The approach relies on the fact that if a gene is conserved over time within multiple strains it gives confidence they will not be lost in the near future and hence are ideal for diagnostic targets. These “core unique” proteins have related sequences in all selected organisms (in this case all available strains of *Bacillus anthracis*) but not in other related organisms. An initial total of 327 proteins unique to the *Bacillus anthracis* proteome were identified using the CUPID and UniProtKB version 13.0. (*Bacillus anthracis* strain Ames isolate Porton, *Bacillus anthracis* strain Ames ancestor, *Bacillus anthracis* strain Sterne were compared to twelve other genomes in the *Bacillus* genus). The two closest relatives of *Bacillus anthracis* as determined by CUPID are *Bacillus thuringiensis* and *Bacillus cereus*. The species most closely related to the selected organism is based on the best BLAST hits of its entire proteome [54]. One needs to be careful in choosing the diagnostic targets that these two non-pathogenic organisms are not being detected.

**Figure 6:**
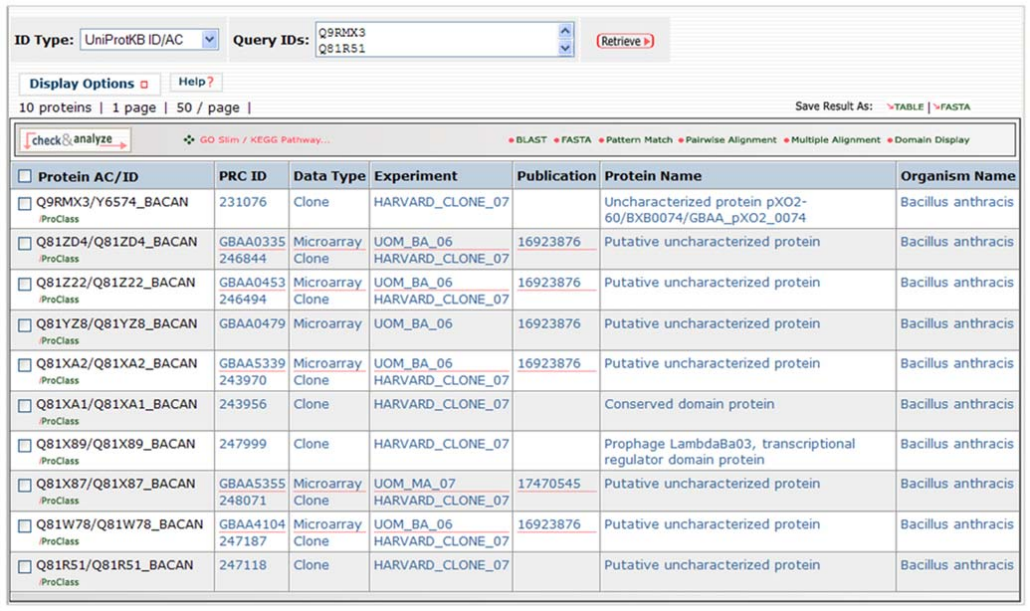
Ten potential diagnostic markers for pathogenic strains of *Bacillus anthracis*. The prioritized protein list was obtained by computationally screening potential targets using CUPID [54], PRC data and other computational means described in the text.

The initial list of 327 was refined to identify “core unique” proteins that are 100 amino acids or more in length. The 100 residue cutoff was used to ensure that the target list consisted of proteins that are real (short proteins might not be real) and are unique, as identification of homologs for short proteins is not trivial [55]. This resulted in a list of 21 “core unique” proteins in the pathogenic strains. It is possible that the proteins found may have homologs in other organisms which were undetected by CUPID because the genes were not annotated as open reading frames To confirm their uniqueness, the 21 proteins were screened for significant regions of similarity at the DNA level (either pseudogenes or unannotated genes) using tBLASTn against the NCBI nr database. Using NCBI's nr which is produced independently of similar, but not identical, sources as iProClass also helps assure no sequences were missing from our warehouse. This additional analysis resulted in a total of 10 *Bacillus anthracis* specific proteins proposed as high-quality targets for development of diagnostic probes. We then supplemented this information with data from the PRC projects and Master Protein Directory to create a matrix of information (Figure 6). Six of the ten targets have data from the University of Michigan PRC showing that they were differentially expressed in published microarray experiments. Nine of the ten are available as clones produced by the Harvard Institute of Proteomics. A search of all the microarray data from the University of Michigan (using http://proteinbank.vbi.vt.edu/ProteinBank/p/search/searchproteins.dll) showed that the four proteins not differentially expressed (listed as clones only in Figure 6) were still constitutively expressed well above background in all studies (not shown). Either the proteins or the DNA coding for these proteins can be used to develop and test pathogen detection systems.

All the “core unique” proteins detected in this study lacked meaningful functional annotation (i.e., were annotated simply as “uncharacterized protein”), which is not surprising as such unique proteins are not easy to characterize. One protein identified as a target is a remnant of a prophage protein. Such proteins are well known to be related to virulence [56]. Another protein is from the pXO2 plasmid. A similar approach was taken for *Salmonella* species using CUPID and public PRC data and several candidate diagnostic proteins are currently being validated in the laboratory (data not shown).

## Discussion

A systems approach to biology or medicine requires the sharing, integration and navigation of large and diverse experimental data sets to develop the models and hypotheses required to make new discoveries and to develop new treatments. To date this has most often been done with selected research data or within an institution or program where common instrumentation and methods make standardization of experimental practices and data management easier to achieve [57], [58], [59]. Alternative approaches require a reanalysis of all the data by a common methodology as has been done in some data repositories [60], [61] or assigning some common statistical metric to all data of a certain type to allow functional coupling [62]. These approaches are all potentially useful, but practically difficult to achieve on a large scale with heterogeneous data. The protein-centric approach we employed is a relatively simple, yet powerful and practical, approach to integrate and navigate diverse sets of omics data in a manner useful for systems biology. Proteins are often the biologically functional elements in cellular networks; thus, many types of data can be mapped to and through proteins as a common biological object.

The lightweight data warehouse approach used for the MPD proved useful in practice, especially with large datasets as its simple design and schema allows greater flexibility to add new data types and to modify search and analysis capabilities. Similar lightweight approaches and schemas designed to optimize queries have been shown useful in integration of genomic data [63], [64]. The main drawback of this approach is that the warehouse does not contain all the data. However, this is rarely a problem if the data are available in some other data resource optimized for that particular data type and if some upfront analysis of the user's needs for query and analysis options is performed. For example, our use case analysis suggested that for microarray and mass spectrometry data, individual raw intensities, machine-specific parameters and most calculated numerical values were not required for general queries and analysis across the combined data as these values were only comparable between the particular analysis performed in one lab. As a result, most numerical values were not included in the MPD for the default search but are accessible for display via hyperlinks to our Protein Data Center or FTP site. However, if a new attribute appear or users request searches on a particular value omitted from the warehouse, adding it is a relatively simple matter of adding new data columns. For instance, in example II our combination and analysis of mass spectrometry and protein interaction data, we could include peptide counts directly in the MPD for immediate download instead of retrieving them from the ftp files. Of course, no one approach can be perfect, as in biology and research there always seems to be exceptions and new data and multiple approaches need to be accommodated.

Efforts to standardize reporting requirements, vocabularies and develop common XML data formats for sharing data are welcome and can greatly ease the transfer and automated processing of a particular data type. However the current standards do not necessarily guarantee integration as the problems of reconciling gene and protein identifiers as well as differences in experimental methodology remain. We investigated and employed a few common data standards and ontologies in developing the Biodefense Proteomics Resource. We provided some data using mzData [65] and MAGE-ML [66] but also provided original data-specific text files for download. We found that several ontologies to describe experimental methods were useful but incomplete and focused on higher eukaryotes and thus did not yet contain terms needed for microbial pathogens. Most useful was the Gene Ontology [67] which has been widely adopted to annotate and classify large scale results and can be used for searching and classification in the MPD.

Here we have presented some unique examples to illustrate benefits, as well as the difficulties, associated with integration of a very diverse set of omics research data across different data types, laboratories and organisms. We illustrated with three examples how potential therapeutic and diagnostic targets can be identified from integrated data applying relatively simple and established tools and techniques. We continue to focus on data integration to allow biologists to find relevant data sets for further detailed analysis using the approaches and tools of their choice. In general the analysis of diverse omics data is an area of active research and a number of useful tools are under active development including cytoscape [28], bioconductor [68] and galaxy [69]. In the future a more seamless integration between data repositories and analysis tools such as these would be the most useful approach to add additional analysis options for integrated data.

## Supporting Information

Table S1 Current fields in the Master Protein Directory. Common and data type specific fields are listed. Description of a field's purpose and examples of some content are shown. All fields are text strings. For more information see schema at ftp://141.161.76.88/pub/proteomics_ftp/rc/MD_data/iproclass_mpd.xsd.
(0.08 MB RTF)
