# Supplementary material for: Systems Integration of Biodefense Omics Data for Analysis of Pathogen-Host Interactions and Identification of Potential Targets
Source: PLoS One. 2009 Sep 25;4(9):e7162. doi: 10.1371/journal.pone.0007162 (PMC2745575; doi:10.1371/journal.pone.0007162)
Supplement: Table S1 — Current fields in the Master Protein Directory. Common and data type specific fields are listed. Description of a field's purpose and examples of some content are shown. All fields are text strings. For more information see schema at ftp://141.161.76.88/pub/proteomics_ftp/rc/MD_data/iproclass_mpd.xsd. (0.08 MB RTF) [file pone.0007162.s001.rtf]

MPD Field Name	Description	Example Content	
PRC_ID	Protein/Gene database Identifier used by the PRC	IPI00216318.4	
Center	PRC name or abbreviation	PNNL	
Data_Type	Name for data type	Mass_Spec	
Experiment_number	Internal code to identify the data set	PNNL_MS_09	
Publication	PubMed ID for PRC publications about the dataset	18205298	
MRD_Link_ID	ID used to link data to a related reagent. 	Q9ZHW2	
View_info_from_PRC	ID used in a URL to link to a PRC database	TgTigrScan_3794	
			
Data Type Specific Fields			
Clone_info			
   Locus_ID	Locus tag from PRC of cloned ORF 	VC1548	
   Vector	Name of cloning vector	pDonr221	
   Insertion_Point	Recombination site, Restriction enzyme sites or nucleotide position of cloned insert	5' attB1/L1 -> attB2/L2 3'	
Interaction info			
   Bait_AC	iProClass accession of Bait (binding domain) protein	Q80HW9	
   Prey_AC	iProClass accession of Prey (acceptor domain) protein	Q96E22	
   Interaction_ID	PRC unique ID for interacting pair	15369909	
Mass_spec_info			
  Expression_Condition	Summary description of key experimental conditions used. Uses controlled vocabulary.	Macrophage, N-negative, Infection Salmonella typhimurium, strain 14028, 4 hour	
   Expression_Status	Summary description of result seen under a particular expression condition, i.e. protein/gene showed an “increase” or “decrease” was “present” or “absent”. Uses controlled vocabulary.	Present	
Comment	Annotated comment about a result from a PRC publication. Uses a controlled vocabulary	Induced after infection	
Microarray_info 			
  Expression_Condition	Summary description of key experimental conditions used. Uses controlled vocabulary.	Macrophage RAW264, Uninfected, Plasma membrane	
   Expression_Status	Summary description of result seen under a particular expression condition, i.e. protein/gene showed an “increase” or “decrease” was “present” or “absent”. Uses controlled vocabulary.	Present	
Comment	Annotated comment about a result from a PRC publication. Uses a controlled vocabulary	Induced after infection	
Structure_info			
   AA_Range	Location of structural domain within larger protein sequence	3837-3919	
   pdb	PDB code for x-ray or NMR domain structure	1YSY	
